# Testing Paradigms of Ecosystem Change under Climate Warming in Antarctica

**DOI:** 10.1371/journal.pone.0055093

**Published:** 2013-02-06

**Authors:** Jessica Melbourne-Thomas, Andrew Constable, Simon Wotherspoon, Ben Raymond

**Affiliations:** 1 Antarctic Climate and Ecosystems Cooperative Research Centre, University of Tasmania, Hobart, Tasmania, Australia; 2 Department of Sustainability, Environment, Water, Population and Communities, Australian Antarctic Division, Kingston, Tasmania, Australia; 3 Institute for Marine and Antarctic Studies, University of Tasmania, Hobart, Tasmania, Australia; Institut Pluridisciplinaire Hubert Curien, France

## Abstract

Antarctic marine ecosystems have undergone significant changes as a result of human activities in the past and are now responding in varied and often complicated ways to climate change impacts. Recent years have seen the emergence of large-scale mechanistic explanations–or “paradigms of change”–that attempt to synthesize our understanding of past and current changes. In many cases, these paradigms are based on observations that are spatially and temporally patchy. The West Antarctic Peninsula (WAP), one of Earth’s most rapidly changing regions, has been an area of particular research focus. A recently proposed mechanistic explanation for observed changes in the WAP region relates changes in penguin populations to variability in krill biomass and regional warming. While this scheme is attractive for its simplicity and chronology, it may not account for complex spatio-temporal processes that drive ecosystem dynamics in the region. It might also be difficult to apply to other Antarctic regions that are experiencing some, though not all, of the changes documented for the WAP. We use qualitative network models of differing levels of complexity to test paradigms of change for the WAP ecosystem. Importantly, our approach captures the emergent effects of feedback processes in complex ecological networks and provides a means to identify and incorporate uncertain linkages between network elements. Our findings highlight key areas of uncertainty in the drivers of documented trends, and suggest that a greater level of model complexity is needed in devising explanations for ecosystem change in the Southern Ocean. We suggest that our network approach to evaluating a recent and widely cited paradigm of change for the Antarctic region could be broadly applied in hypothesis testing for other regions and research fields.

## Introduction

The West Antarctic Peninsula (WAP) is a global ‘hotspot’ for atmospheric warming, with increases in mean temperatures in the order of 1°C per decade since the middle of last century [1], [2] and associated cryospheric changes [3], [4]. As elsewhere in the Southern Ocean, this region experienced near extirpation of Antarctic fur seals and whales over the last two centuries but populations are now recovering [5], [6]. The region supports large and diverse populations of marine mammals and birds dependent on Antarctic krill, which is the dominant prey species [7]. A fishery for krill has been expanding in the region since the 1980s and, more recently, access to fishing grounds during winter has increased [8]. Moreover, the current ecosystem effects of depleted demersal fish stocks are unclear [9], [10]. Not surprisingly, this has been an area of particular interest for exploring ecological responses to rapid environmental change [7], [11], [12], [13].

A recently proposed mechanistic explanation for changes in pygoscelid penguin populations across the WAP region centers on a chronology of change, beginning with the substantial reductions in marine mammals followed by overfishing of finfish in the 1970s and more recent effects of changing availability of krill prey and climatic conditions [11]. Specifically, Trivelpiece et al. [11] argue that there is sufficient empirical evidence to indicate populations of both Adélie penguins (*Pygoscelis adeliae*) and chinstrap penguins (*P. antarctica*) have declined in this region since the late 1970s. When viewed in conjunction with other conclusions of change in the region, these population declines are attributed to the combined effects of increased competition for krill prey (from the expanding krill fishery and from recovering top predators) and unfavorable climatic conditions. The presented evidence indicates a potential chain of linked events, or mechanisms, that ultimately give rise to the decline in penguin populations. However, other ecosystem interactions can be identified that may give rise to positive and negative feedbacks [12], [13], [14], which potentially make it difficult to draw conclusions on causes of penguin declines.

Complex ecological networks – such as Antarctic marine ecosystems – are characterized by feedback cycles, nonlinear responses and indirect effects on the propagation of a disturbance following perturbation. These features mean that network-level responses can be very different to what might be expected from the simple aggregation of a chain of species-level responses. Network modeling is a valuable tool for capturing feedback effects and for making predictions regarding responses to a perturbation. Qualitative network modeling [15], [16] is based on an analysis of the *direction* of interactions (i.e. positive or negative effects) between network components. Because this method does not rely on quantitative data, it is an attractive approach for rapid model formulation and hypothesis testing regarding ecosystem structure and function.

We used a qualitative network modeling approach to test the mechanistic explanation for changes in penguin populations, and to assess the potential for broader, foodweb-level effects of regional warming for the WAP. Specifically, we constructed and analyzed a set of three models of differing levels of complexity that capture key variables and processes for the WAP region as described by Trivelpiece et al. [11] and in the broader literature. In the following sections we describe the methods for constructing and analyzing our set of models. We then assess and compare the ability of these models to capture documented changes in penguin populations, and suggest important linkages and variables to be targeted in future studies to further inform understanding of WAP ecosystem function and change. While qualitative network modeling has been applied previously to explore species- and community-level responses to perturbation for a range of marine and terrestrial systems (e.g. [15], [17], [18], [19]), our application of the approach for the WAP demonstrates how network analysis could be more broadly applied to model paradigms of regional-scale ecosystem change. Importantly, our analysis is not intended as an exhaustive, quantitative evaluation of the mechanisms of change in the WAP region, but rather as a demonstration of how network models can be used to critically evaluate assumptions about the structure and function of ecological networks and to identify key hypotheses for testing in the future.

## Materials and Methods

### A Network Approach to Hypothesis Testing

**Figure 1:**
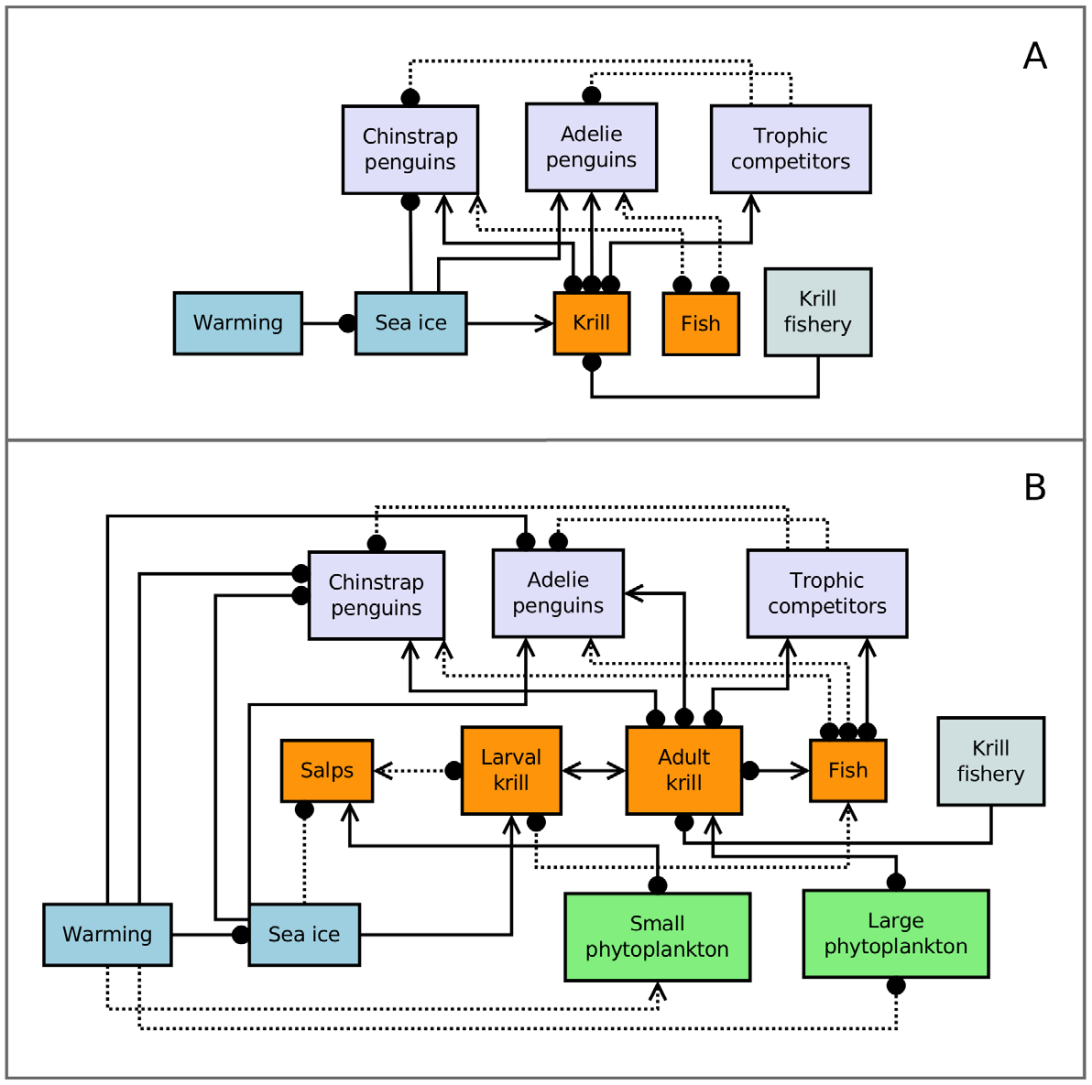
Qualitative network models for the West Antarctic Peninsula (WAP) ecosystem. Arrows and filled circles represent positive and negative effects, respectively, exerted by one model component on another. Dashed lines represent uncertain interactions. (A) Prey-limitation model as described by Trivelpiece et al. [11]. (B). Extended network model with additional planktonic groups of particular ecological significance for the region [13]. All model components have a limiting (negative) self-interaction, but for clarity these are not shown. Detailed descriptions of linkages in the extended model are provided in the supporting information.

Network models are composed of variables and linkages (equivalently referred to as nodes and edges). In ecology, model linkages conventionally represent trophic interactions (i.e. foodweb models), but more recent implementations extend to other ecological interactions such as competition, habitat dependencies and environmental drivers. Mathematically, the analysis of network models is built on the formalisms of graph theory and matrix algebra; specifically, analysis of the community matrix [20]. Qualitative network analysis (developed from so-called ‘loop analysis’; [21]) predicts the response of a model system to a press perturbation (defined as a sustained shift in the *per capita* growth rate of a population [22]), from the inverse of the qualitatively specified community matrix, where only the signs of matrix elements are specified [23]. As only the signs are considered, qualitative models can be represented by signed directed graphs (or signed digraphs, e.g. Fig. 1). The variables in a signed digraph represent modeled populations or processes, and the linkages (or directed edges) represent the non-zero matrix elements. With this scheme, the sign structure of the community matrix is determined by the signed adjacency matrix of the directed graph (see [16]). This type of approach is appropriate for applications where there is uncertainty about the particular mathematical forms of the interactions in the system (i.e. parametric uncertainty). Knowledge of the system structure (i.e. which nodes are present, and how they are connected) and the signs of the interactions is sufficient to allow the user to make qualitative predictions about the effects of perturbations to the system.

**Figure 2:**
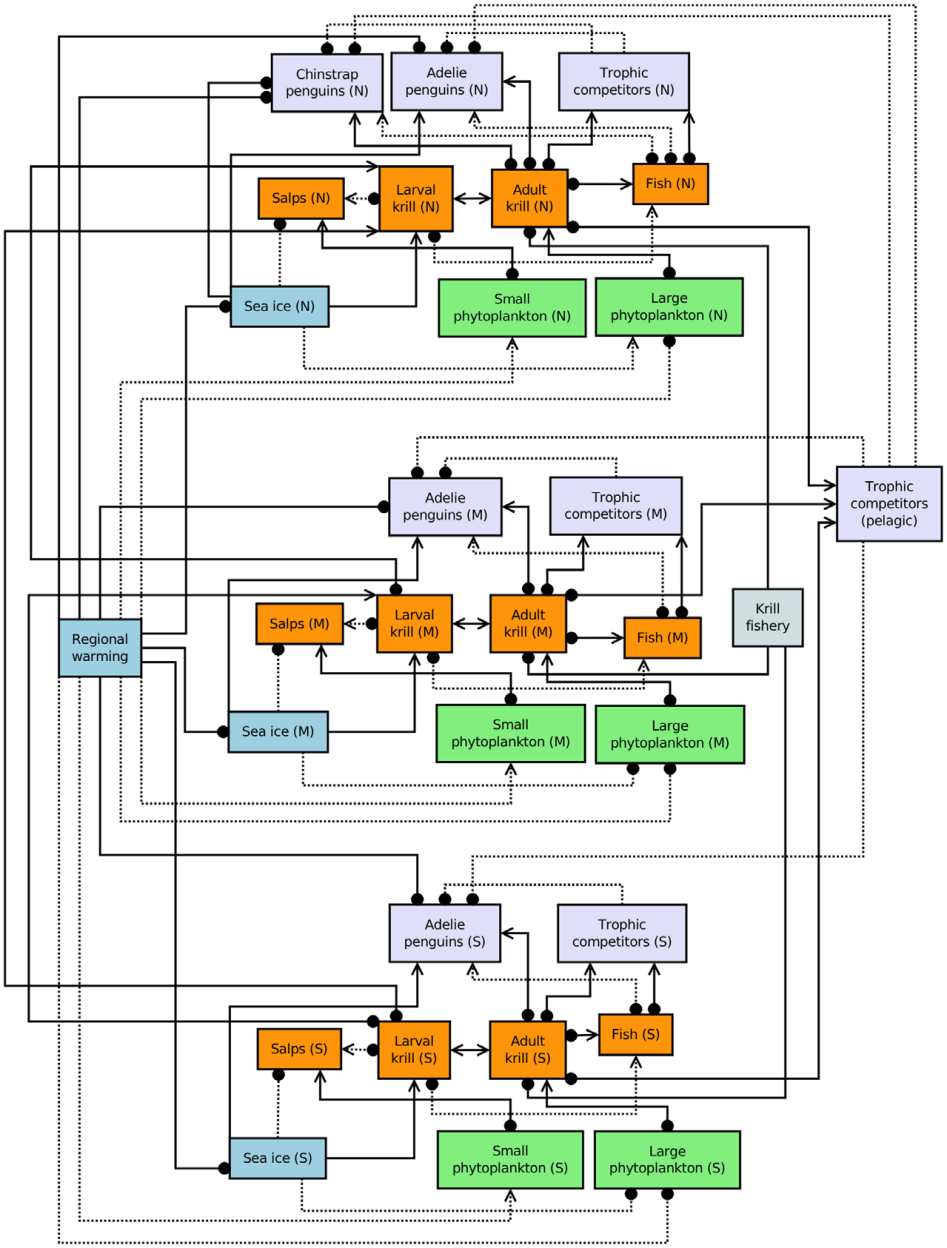
Spatial implementation of the extended network model for the WAP ecosystem (Fig. 1B). Northern (N), middle (M) and southern (S) subregions correspond approximately with the South Shetland Islands, the Palmer Long-Term Ecological Research Program area and Marguerite Bay, respectively. The model includes south to north transport of larval krill [14], and pelagic foragers as trophic competitors with Adélie and chinstrap penguins. Chinstrap penguins are restricted to the northern subregion [30], [31]. All model components have a limiting (negative) self-interaction, but for clarity these are not shown.

In some applications, however, uncertainty can be even more pervasive, and extend to the structure of the system itself. That is, it may not be clear whether or not two elements in the system interact, and therefore whether the associated interaction should be included in the model. Recently developed simulation approaches for qualitative network modeling [15], [16] enable structural and parametric uncertainty to be simultaneously addressed. This is done by analyzing a range of possible model configurations (with and without uncertain interactions; see dashed lines in Figs. 1 and 2). Each model configuration is simulated a number of times using randomly assigned interaction weights (for both certain and uncertain interactions), each drawn from a uniform distribution. Model stability is tested at each step by examining the eigenvalues of this quantitatively specified community matrix, and results from stable configurations are collated. An optional validity criterion can also be applied, which requires that the model be able to reproduce a previously observed response to a known perturbation scenario. Models that are unable to reproduce this particular response are discarded. Simulation results from all stable, valid models are aggregated to provide estimates of the likelihood of different outcomes under particular press perturbation scenarios. The use of randomly assigned weights effectively allows the analyses to explore the effects of parametric uncertainty, and the range of model configurations the structural uncertainty. Full descriptions of the simulation approach used here, together with formal descriptions of model representation, are given in Melbourne-Thomas et al. [16].

We emphasize two features of our application of qualitative network modeling for evaluating paradigms of change for the WAP region. First, our approach does not involve statistical evaluation of models against data. Instead, we make direct comparisons of qualitative outcomes from models with expectations regarding the direction of change for key model variables. Second, the construction and analysis of our WAP ecosystem models requires only assumptions that relate to ecological processes (i.e. which network components interact with which others, and in what qualitative manner). No assumptions about historical changes in populations are required. This strengthens the value of our modeling approach as an independent test of the hypothesized drivers of ecological change in the WAP region. This feature of our approach is pertinent given that there is little direct evidence for the changes in krill availability and abundances of competing top predators postulated by Trivelpiece et al. [11]. Specifically, there is weak, if any evidence for increases in krill abundance under whaling (indeed krill populations may have been larger in the early 1900s than they are now; [24]), and no consistent trend in the population sizes of major krill predators that might compete with Adélie and chinstrap penguins [6], except for Antarctic fur seals (*Arctocephalus gazella*) in the northern WAP [5] and humpback whales (*Megaptera novaeangliae*) [25].

### Network Modeling for the WAP

We formulated three alternative representations of ecosystem linkages for the WAP (Figs. 1 and 2). Models were constructed as signed digraphs in the drawing program Dia (http://live.gnome.org/Dia). This construction process allows interactions to be characterized in terms of both their sign (positive or negative) and certainty. Solid lines indicate interactions for which we believe there is firm evidence for their importance to the associated model components. Dashed lines indicate uncertain interactions, which we believe to be plausible but are not yet confirmed as important.

The first of our three models (Fig. 1A) replicates key processes described by Trivelpiece et al. [11] in their mechanistic explanation for observed changes in the WAP region. This model is referred to as the ‘prey-limitation’ model. Our second model (Fig. 1B) captures additional trophic complexity, specifically the importance of small and large phytoplankton at the base of the food web, and the potential effects of warming on these groups [13]. This ‘extended’ model resolves Antarctic krill (*Euphausia superba*) into larval and adult stages, and hence captures the importance of sea-ice habitats for larval krill [26]. The model also includes salps (specifically *Salpa thompsoni*), which are a zooplankton group of significant interest for the WAP [13], [27] and the Southern Ocean in general [24], [28], particularly in the context of climate-related changes to sea ice.

The sea ice variable in both the prey limitation and extended models captures the effects of the spatial extent and temporal duration (i.e. the difference between timing of advance and retreat) of sea ice on other model components (see Table S1). Recent warming for the WAP region has been linked to decreases in both these sea-ice descriptors [3], [4]. The trophic competitor variable in these models represents marine mammals found in the region that have krill as a significant component of their diets (e.g. fur seals and baleen whales; [29]), and could hence potentially compete with penguins for prey. This competition is assumed to be one-way interference competition. Finally, ‘large’ and ‘small’ descriptors for phytoplankton groups in the extended model refer to cell sizes, with the large group being dominated by diatoms, and the small group being dominated by cryptophytes [12], [13]. Detailed descriptions of model linkages in our extended WAP model are provided Table S1.

Our third model (Fig. 2) is a spatially resolved version of our extended model. This ‘spatial’ model replicates the interactions captured in the extended model for southern, middle and northern subregions of the WAP (which correspond approximately with Marguerite Bay, the Palmer Long-Term Ecological Research Program area and the South Shetland Islands, respectively). Additional features of the spatial model are (i) pelagic foragers as trophic competitors with Adélie and chinstrap penguins, (ii) northward transport of larval krill [14], (iii) range limitation for chinstrap penguins (this group is only represented in the northern model subregion; [30], [31]), and (iv) differential effects of sea ice on large phytoplankton; specifically, a positive (but uncertain) effect of sea ice on large phytoplankton in the northern subregion, but a negative (uncertain) effect in middle and southern subregions [12].

Our three network representations – the prey-limitation model, extended model, and spatial model – were used to evaluate probabilities for qualitative responses (increase, no change or decrease) of key ecosystem components, including Adélie and chinstrap penguins, to a region-wide warming trend and an increase in the WAP krill fishery. We used previously described simulation and analysis methods for qualitative network models with uncertain linkages [15], [16]. All potential model configurations (i.e. with all possible combinations of uncertain linkages) were examined. The qualitative responses (i.e. increase, decrease or no change) of model components to a press perturbation scenario of increased regional warming and an increase in the krill fishery were aggregated across all stable model configurations (where all interaction weights, both certain and uncertain, were randomly sampled from a uniform distribution). R code and Dia models used in our analyses are provided in Supplementary code S1.

### Sensitivity Analysis

We examined the sensitivity of model predictions to the choice of (i) number of simulations, (ii) perturbed model variables, and (iii) constraints on model selection. For case (i) we compared model predictions from 10^2^, 10^3^ and 10^4^ simulations (where 10^4^ is the default value). In case (ii) we compared the effect of perturbing the krill fishery and regional warming concurrently with model predictions from perturbing each of these variables separately. Finally, for case (iii) we examined the effects of constraining model configurations to meet two conditions: (a) decreases in penguin populations (both Adélie and chinstrap penguins) under a combined scenario of warming and an increase in the krill fishery, and (b) an increase in adult krill in response to decreased populations of trophic competitors (the ‘krill surplus’ hypothesis implicitly assumed in Trivelpiece et al.’s [11] explanation for past changes in penguin populations).

As indicated above in our description of simulation approaches to qualitative network modeling, it is possible to constrain allowable models using validation criteria. That is, a candidate model must reproduce a previously documented response to a known perturbation before it is included in further analyses [15], [16]. In the case of our WAP model, there is no clear choice of non-trivial validation criteria. Pygoscelid penguin population responses are not suitable as validation criteria for our main set of analyses because our aim is to assess the ability of models to reproduce documented changes in these populations. Furthermore, no non-trivial responses to press perturbation scenarios have been documented for any other taxonomic group in the WAP region. Case (iii) of our sensitivity analysis is therefore intended to assess the sensitivity of model outcomes to constraints on uncertain model linkages, but observed outcomes are not suited to further interpretation.

Comparisons of model predictions for scenarios in cases (i) to (iii) were made using principle components analysis (PCA) of the proportions of simulations indicating a negative change for each model variable. Results are presented for the most complex system in our model set, the spatial model. While this is not intended as an exhaustive test of model sensitivity, it explores key assumptions that are specific to our example analysis for the WAP ecosystem.

## Results and Discussion

### Responses to Change

**Figure 3:**
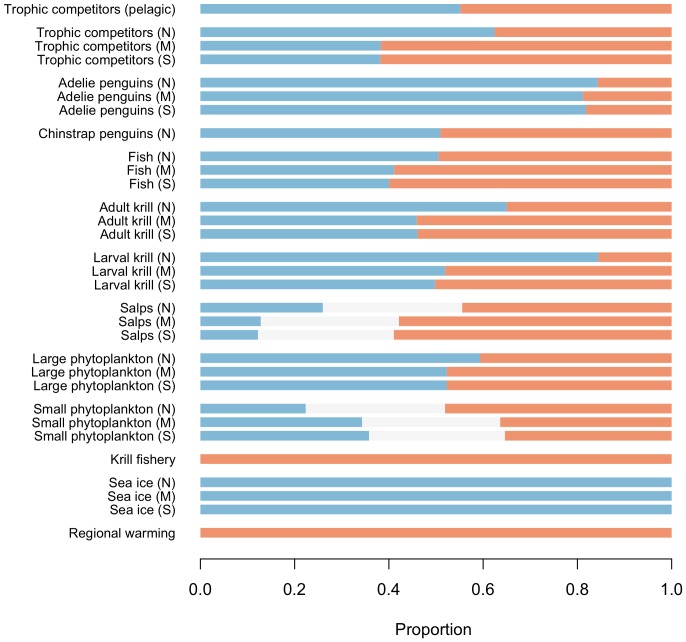
Proportional outcomes from 10^3^ simulations for the spatial WAP model under a scenario of increased regional warming and an increase in the krill fishery. Blue represents a negative change, grey is no change, and orange is a positive change. Results indicate a higher propensity for negative change in the northern subregion (N) as compared to middle (M) and southern (S) subregions across a range of taxa.

All three of our qualitative network representations of the WAP ecosystem provide only limited evidence to support documented decreases in chinstrap penguin populations in response to climate-driven change. Under the prey-limitation model (Fig. 1A), only 26% of simulations indicated a decrease in chinstrap penguins under a scenario of regional warming and an increase in the krill fishery (Table 1). Even for the case where increases in trophic competitors were also included in the perturbation scenario, less than half of the simulations (40%) indicated an increase for this group. The only means to achieve >50% support for a decrease in chinstrap penguins under the prey-limitation model was to delete model linkages that represent the direct effects of sea ice on (i) chinstrap penguins or (ii) both chinstrap and Adélie penguins (this yielded predicted decreases of chinstraps in 71% and 84% of simulations, respectively). Both the extended and spatial models (Figs. 1B and 2) gave ambiguous predictions for the response of chinstrap penguins to recent change in the WAP region (i.e. approximately equal support for positive versus negative change under the perturbation scenario; Table 1, Fig. 3). These results indicate that while some model configurations supported conclusions of decline in Chinstrap penguins, the combination of mechanisms needed to result in a decline is ambiguous at present and needs further development.

**Table 1:** Proportional outcomes (expressed as percentages) from 10^3^ simulations for the prey-limitation model (Fig. 1A) and the extended model (Fig. 1B) under a scenario of increased regional warming and an increase in the krill fishery.

	Prey-limitation model		Extended model	
	–	+	–	+
Adélie penguins (%)	89.8*	10.2	91.8*	8.2
Chinstrap penguins (%)	26.3	73.7*	54.7	45.3
Krill (%)	86.6*	13.4	62.4	37.6
Trophic competitors (%)	86.6*	13.4	56.7	43.2

Results are summarized as the percentage of simulations under which a subset of modeled groups (Adélie and chinstrap penguins, krill and trophic competitors) underwent negative or positive change. Cases of clear model support for an increase or decrease under the perturbation scenario (i.e. >70% of predictions) are asterisked. The full set of outcomes from these analyses is provided in Figs. S1 and S2.

Outcomes for chinstrap penguins differed between the prey-limitation and extended models. This may be explained in part by a dominating effect of sea ice/penguin interactions in the prey-limitation model. That is, decreasing sea ice had a positive effect on chinstrap penguins due to their requirement for ice-free foraging areas during winter, but a negative effect on Adélie penguins, who favor pack-ice habitat in winter [11], [30]. Deleting both of these effects from the prey-limitation model led to the highest predictions for decreases in chinstrap penguins, most likely because of increased competition for krill prey between penguin species under this model configuration. In the extended model, other feedback processes counteract the direct effects of sea ice, leading to ambiguous predictions for changes in chinstrap penguin populations.

Discrepancies between our model-based results and changes reported by Trivelpiece et al. [11] may relate to expectations for population-wide responses. Our model assumes population-wide rather than colony-specific effects. Trivelpiece et al. [11] did not include some data for chinstrap penguins in their analyses (specifically, data from Admiralty Bay and from the Palmer Long-Term Ecological Research Program). Nevertheless, while previous authors have reported increases in chinstrap populations since the 1970s [29], [32], [33], results from local-scale studies [34], [35] and a recent integration of region-wide data [31] indicate declines in local and population-weighted averages for chinstrap penguin colonies. Importantly, these more recent findings highlight spatial and inter-colony variability in population trends; ambiguity in predictions from our extended model is consistent with this observation.

In support of excluding a direct linkage between sea ice and pygoscelid penguins, we note that Lynch et al. [31] found no statistically significant correlation between November (spring) sea ice concentration and chinstrap (or Adélie) penguin population change. However, the effects of other potentially important sea ice variables (e.g. duration and timing of advance and retreat) were not included in their analyses. The development of a more coherent model relating changes in pygoscelid penguin populations to sea ice decline in the WAP region is clearly an important area for future observational and modeling studies.

In contrast to our results for chinstrap penguins, all three models indicated decreases in Adélie penguins in response to the combined impacts of regional warming and an increase in the krill fishery (in the order of 80 to 90% of simulations from all three models indicated a decrease in Adélie penguins under this scenario; Table 1, Fig. 3). Results for other taxa and functional groups differed between models. Eighty-seven percent of simulations for the prey-limitation model indicated decreases for both trophic competitors and krill under our combined warming and fishing scenario (Table 1). This was in contrast with more ambiguous predictions for these groups from both the extended and spatial models (i.e. approximately equal support for both increases and decreases in trophic competitors and krill; Table 1, Fig. 3). Ambiguity in model predictions for trophic competitor responses to regional warming is consistent with real world uncertainty about the direction of recent changes from empirical studies [6], [29]. Future assessments will need to address how the availability of krill to primary colonies of interest may be affected, along with interactions with potentially competing predators, rather than modeling solely on the basis of potential interactions among populations.

Results for krill from our extended model are somewhat unexpected, given existing evidence for declines in krill abundance in response to declining sea ice in the WAP region [24]. However, we note that a high proportion of simulations indicated a decrease in larval krill under our perturbation scenario (86% for the extended model and 51–84% for the spatial model). Based on these findings we highlight the need to (a) combine foodweb modeling approaches with more highly resolved stage-structured models (e.g. [36], [37]) to gain a full picture of krill meta-population dynamics, and (b) continue to evaluate the status and trends of krill stocks across the WAP region to extend and verify current knowledge [24], [38], [39].

Embedding network processes in a spatial context (which has not previously been achieved using qualitative models of marine ecosystems), suggests spatial differences in the responses of key taxa to climate-driven warming in the WAP region. Results from our spatial model indicated higher proportions of potential negative changes in the northern WAP subregion, as compared with middle and southern subregions, for trophic competitors, penguins, fish, adult and larval krill, salps and large phytoplankton (Fig. 3). Interestingly, the reverse was true for small phytoplankton. Model results for both salps and small phytoplankton indicated a relatively high probability of zero change (in the order of 30%) under our perturbation scenario. This is in contrast to all other modeled taxa and functional groups that exhibited only positive or negative change in simulations.

The reasons for modeled spatial differences in susceptibility to regional warming and an increase in the krill fishery are worthy of further examination. In our spatial model, feedback effects may be amplified through northward transport of larval krill. Alternatively, spatial differences may be related to bottom-up controls of large phytoplankton, specifically, our inclusion of a positive (but uncertain) effect of sea ice on large phytoplankton in the northern subregion, but a negative (uncertain) effect in middle and southern subregions (which translates to a negative effect of regional warming on large phytoplankton in the north as compared with a potential positive effect in the south). This difference is based on recent evidence that decreased summer sea-ice extent is favorable to phytoplankton blooms in middle and southern subregions of the WAP, but leads to reduced photosynthesis in the north (both these effects are most pronounced for large phytoplankton cells; [12]). Given uncertainties about this and other key processes associated with change in Antarctic marine ecosystems, our qualitative modeling approach provides a means to identify priorities for future research.

### Model Sensitivity

**Figure 4:**
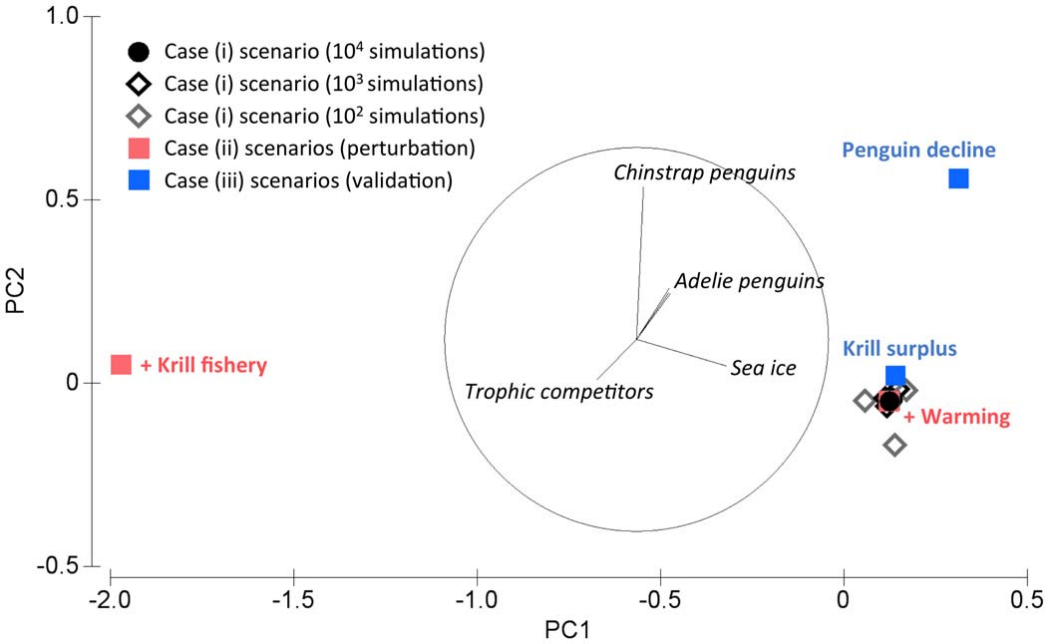
Principal components ordination of model predictions (proportion of simulations indicating a negative change for each variable in the spatial model) from sensitivity analyses. Eigenvectors (with magnitudes >0.2) are represented as a bioplot. 96% of the total variance is captured by PC1 (88%) and PC2 (8%). Results from varying the number of simulations used in qualitative network analyses (case (i) scenarios) are shown in black and grey; the filled grey circle represents the main perturbation scenario examined (concurrent increases in the krill fishery and warming) and default number of simulations (10^4^). Pink squares represent scenarios where the krill fishery and warming were perturbed separately (case (ii) scenarios), while blue squares represent scenarios where models were constrained to meet specific criteria (case (iii) scenarios, as described in the main text).

Results from sensitivity analysis for the three cases described in our methods, using the most complex of our three models (Fig. 4), indicate that for the first case (number of simulations) variability in predictions increased as the number of simulations decreased (as would be expected). More interestingly, sensitivity results for the second case (perturbed model variables) demonstrate the differential effects of the two components of our main perturbation scenario. Specifically, network-level responses to a combined scenario of increases in the krill fishery and increased warming were dominated by the effect of warming. An increase in the krill fishery alone was associated with a decrease in trophic competitors, while scenarios of increased warming were characterized by decreases in sea ice and Adélie penguins and resulted in model outcomes that were almost identical to our main scenario of an increase in both fishing and warming (Fig. 4).

As discussed in our methods section, the network analysis approach adopted here allows for restrictions to be imposed on model configuration based on prior expectations for model behavior (case (iii) in our methods). While there is no clear choice of (non-trivial) validation criteria for our WAP model (for reasons discussed in the methods section above) we did explore the sensitivity of model predictions to imposing (a) decreases in penguin populations under a combined scenario of warming and an increase in the krill fishery, and (b) an increase in adult krill in response to decreased populations of trophic competitors. Model predictions under restriction (a) are distinct from model results under the main scenario in having a higher proportion of predictions for decreases in penguin populations (but not in terms of other modeled groups). Model predictions under restriction (b) are similar to the main scenario, but with a slightly higher proportion of predictions for penguin population declines (Fig. 4).

### Caveats and Limitations

The models presented here are intended to be minimum realistic (*sensu* Fulton et al.; [40]) for the purpose of evaluating mechanisms of change for key WAP taxa, particularly penguins. Clearly these models are not the only possible representations of the WAP system. Our models intentionally do not resolve coastal versus offshore differences in ecological processes [41]; a useful extension to our study would be to consider alternative model formulations for these different habitats and to address the importance of ecosystem effects at the meso-scale (such as those discussed by Trathan et al. [42] for macaroni penguins at South Georgia). We excluded groups such as crystal krill (*Euphausia crystallorophias*) and copepods that are of some interest for the region [27], [43], [44], because we found during exploratory analyses that they did not play a significant role in network level dynamics. We note that some authors suggest that populations of sea-ice dependent Antarctic silverfish (*Pleuragramma antarcticum*), which are a potential prey item for penguins, may have declined in the WAP region [45]. However, evidence for this decline appears to be limited to observations of decreased proportions of silverfish in Adélie penguin diets [46]. A decline in the model variable representing fish was therefore not included in our scenarios.

We also note that the models we elaborate here did not resolve phenological changes that are likely to be important in determining the responses of pygoscelid penguins to environmental change [47], [48]. Finally, the nature of our approach (qualitative network modeling) dictates that model linkages are not directly parameterized, and that predictions for responses to change do not capture relative magnitudes of responses, only their direction. Nevertheless, our approach and findings strongly support the need to consider system-level feedback processes in evaluating mechanistic explanations for past and present processes in Southern Ocean ecosystems.

### Conclusions

There are significant uncertainties associated with the mechanisms behind past and current patterns of change in Antarctic marine ecosystems. It is important to revisit and re-evaluate established paradigms of change using a combination of empirical and modeling approaches. We demonstrate the use of qualitative network models as a standard approach to evaluate a conceptual mechanistic explanation for changes in penguin populations in the WAP region. The key advantage of this approach is its ability to capture emergent, foodweb level responses that might not be apparent from a species-by-species approach to understanding ecological change. This approach also enables explanations of direct and indirect change amongst a subset of ecological relationships to be tested within the context of the greater network of interactions. While the WAP system is a good candidate for this approach because of its relatively long history of empirical observation, our methods could readily be extended to other Antarctic regions that are experiencing contrasting patterns of change in key physical drivers. We also suggest the broader application of qualitative network modeling as an objective method for testing assumptions regarding system structure and function in ecosystem studies.

## Supporting Information

Figure S1 **Proportional outcomes from 10^3^ simulations for the prey-limitation model (** **Fig. 1A** **, main text) under a scenario of increased regional warming and an increase in the krill fishery.** Blue represents a negative change, grey is no change, and orange is a positive change.
(DOCX)

Figure S2 **Proportional outcomes from 10^3^ simulations for the extended (non-spatial) West Antarctic Peninsula model (** **Fig. 1B** **, main text) under a scenario of increased regional warming and an increase in the krill fishery.** Blue represents a negative change, grey is no change, and orange is a positive change.
(DOCX)

Table S1 Descriptions of model edges (linkages) for the extended WAP model (Fig. 1B, main text). Weights refer to positive ( = 1) and negative ( = −1) effects from one model component to another. Shaded rows indicate uncertain linkages.
(DOCX)

Supplementary code S1 **R code and Dia models used in analyses.**
(ZIP)
