# Supplementary material for: Testing Paradigms of Ecosystem Change under Climate Warming in Antarctica
Source: PLoS One. 2013 Feb 6;8(2):e55093. doi: 10.1371/journal.pone.0055093 (PMC3566216; doi:10.1371/journal.pone.0055093)
Supplement: Figure S1 — Proportional outcomes from 103 simulations for the prey-limitation model ( Fig. 1A , main text) under a scenario of increased regional warming and an increase in the krill fishery. Blue represents a negative change, grey is no change, and orange is a positive change. (DOCX) [file pone.0055093.s001.docx]

Figure S1. Proportional outcomes from 10^3^ simulations for the prey-limitation model (Fig. 1A, main text) under a scenario of increased regional warming and an increase in the krill fishery. Blue represents a negative change, grey is no change, and orange is a positive change.
