# Supplementary material for: Testing Paradigms of Ecosystem Change under Climate Warming in Antarctica
Source: PLoS One. 2013 Feb 6;8(2):e55093. doi: 10.1371/journal.pone.0055093 (PMC3566216; doi:10.1371/journal.pone.0055093)
Supplement: Table S1 — Descriptions of model edges (linkages) for the extended WAP model (Fig. 1B, main text). Weights refer to positive ( = 1) and negative ( = −1) effects from one model component to another. Shaded rows indicate uncertain linkages. (DOCX) [file pone.0055093.s003.docx]

Table S1. Descriptions of model edges (linkages) for the extended WAP model (Fig. 1B, main text). Weights refer to positive (=1) and negative (=-1) effects from one model component to another. Shaded rows indicate uncertain linkages.

| **From** | **To** | **Weight** | **Sources** | **Notes** |
| --- | --- | --- | --- | --- |
| Warming | Sea ice | -1 | [1,2] |  |
| Warming | Chinstrap penguins | -1 | [3] | (a) |
| Warming | Adélie penguins | -1 | [3] | (a) |
| Warming | Small phytoplankton | 1 | [4] | (b) |
| Warming | Large phytoplankton | -1 | [4] | (b) |
| Sea ice | Chinstrap penguins | -1 | [5,6] | (c) |
| Sea ice | Adélie penguins | 1 | [5,6] | (c) |
| Sea ice | Salps | -1 | [7] |  |
| Sea ice | Larval krill | 1 | [8,9] |  |
| Salps | Larval krill | -1 | [10] |  |
| Salps | Small phytoplankton | -1 | [4] |  |
| Larval krill | Salps | 1 | [10] |  |
| Larval krill | Fish | 1 | [11] |  |
| Larval krill | Adult krill | 1 |  | (d) |
| Trophic competitors | Fish | -1 | [12] |  |
| Trophic competitors | Chinstrap penguins | -1 | [5] | (e) |
| Trophic competitors | Adélie penguins | -1 | [5] | (e) |
| Trophic competitors | Adult krill | -1 | [5,12] |  |
| Krill fishery | Adult krill | -1 |  |  |
| Fish | Larval krill | -1 | [11] |  |
| Fish | Adult krill | -1 | [11] |  |
| Fish | Trophic competitors | 1 | [12] |  |
| Fish | Chinstrap penguins | 1 | [5,13,14] | (f) |
| Fish | Adélie penguins | 1 | [5,13,14] | (f) |
| Large phytoplankton | Adult krill | 1 | [4] |  |
| Small phytoplankton | Salps | 1 | [4] |  |
| Chinstrap penguins | Fish | -1 | [5,13,14] | (f) |
| Chinstrap penguins | Adult krill | -1 | [5,15] |  |
| Adult krill | Larval krill | 1 |  |  |
| Adult krill | Trophic competitors | 1 | [5,12] |  |
| Adult krill | Fish | 1 | [11] |  |
| Adult krill | Large phytoplankton | -1 | [4] |  |
| Adult krill | Chinstrap penguins | 1 | [5,15] |  |
| Adult krill | Adélie penguins | 1 | [5,15] |  |
| Adélie penguins | Fish | -1 | [5,13,14] | (f) |
| Adélie penguins | Adult krill | -1 | [5,15] |  |

1. Effect of chick wetting due to increased precipitation or snow-melt in colonies under regional warming
2. Mediated by decreased nearshore salinity due to increased meltwater
3. Chinstrap penguins forage in ice-free water during winter while Adélie penguins favor pack-ice habitat in winter
4. Krill reproduction and recruitment
5. One-way interference competition for krill prey
6. Fish comprise a low percentage of penguin diets (≤5%)

**References**

1. Liu J, Curry JA, Martinson DG (2004) Interpretation of recent Antarctic sea ice variability. Geophysical Research Letters 31: L02205.

2. Smith RC, Stammerjohn SE (2001) Variations of surface air temperature and sea-ice extent in the western Antarctic Peninsula region. Annals of Glaciology 33: 493-500.

3. Chapman E, Hofmann E, Patterson D, Ribic C, Fraser W (2011) Marine and terrestrial factors affecting Adélie­ penguin *Pygoscelis adeliae* chick growth and recruitment off the western Antarctic Peninsula. Marine Ecology Progress Series 436: 273-289.

4. Moline MA, Claustre H, Frazer TK, Schofield O, Vernet M (2004) Alteration of the food web along the Antarctic Peninsula in response to a regional warming trend. Global Change Biology 10: 1973-1980.

5. Trivelpiece WZ, Hinke JT, Miller AK, Reiss CS, Trivelpiece SG, et al. (2011) Variability in krill biomass links harvesting and climate warming to penguin population changes in Antarctica. Proceedings of the National Academy of Sciences 108: 7625-7628.

6. Fraser WR, Trivelpiece WZ, Ainley DG, Trivelpiece SG (1992) Increases in Antarctic penguin populations: reduced competition with whales or a loss of sea ice due to environmental warming? Polar Biology 11: 525-531.

7. Ross R, Quetin L, Martinson D, Iannuzzi R, Stammerjohn S, et al. (2008) Palmer LTER: Patterns of distribution of five dominant zooplankton species in the epipelagic zone west of the Antarctic Peninsula, 1993–2004. Deep Sea Research Part II: Topical Studies in Oceanography 55: 2086-2105.

8. Loeb V, Siegel V, Holm-Hansen O, Hewitt R (1997) Effects of sea-ice extent and krill or salp dominance on the Antarctic food web. Nature 387: 897-900.

9. Nicol S (2006) Krill, currents, and sea ice: *Euphausia superba* and its changing environment. BioScience 56: 111-120.

10. Nishikawa J, Naganobu M, Ichii T, Ishii H (1995) Distribution of salps near the South Shetland Islands during austral summer, 1990–1991 with special reference to krill distribution. Polar Biology: 31-39.

11. Lancraft TM, Reisenbichler KR, Robison BH, Hopkins TL, Torres JJ (2004) A krill-dominated micronekton and macrozooplankton community in Croker Passage, Antarctica with an estimate of fish predation. Deep Sea Research Part II: Topical Studies in Oceanography 51: 2247-2260.

12. Ducklow HW, Baker K, Martinson DG, Quetin LB, Ross RM, et al. (2007) Marine pelagic ecosystems: the West Antarctic Peninsula. Philosophical Transactions of the Royal Society B: Biological Sciences 362: 67-94.

13. Trivelpiece WZ, Trivelpiece SG, Volkman NJ (1987) Ecological segregation of Adelie, Gentoo, and Chinstrap penguins at King George Island, Antarctica. Ecology 68: 351-361.

14. Miller AK, Kappes MA, Trivelpiece SG, Trivelpiece WZ (2010) Foraging-niche separation of breeding Gentoo and Chinstrap penguins, South Shetland Islands, Antarctica. The Condor 112: 683-695.

15. Lynnes AS, Reid K, Croxall JP (2004) Diet and reproductive success of Adélie and Chinstrap penguins: linking response of predators to prey population dynamics. Polar Biology 27: 544-554.
